# Rotavirus NSP1 Requires Casein Kinase II-Mediated Phosphorylation for Hijacking of Cullin-RING Ligases

**DOI:** 10.1128/mBio.01213-17

**Published:** 2017-08-29

**Authors:** Kaitlin A. Davis, Marco Morelli, John T. Patton

**Affiliations:** a Department of Veterinary Medicine, Virginia—Maryland College of Veterinary Medicine, University of Maryland, College Park, Maryland, USA; b Laboratory of Infectious Diseases, National Institute of Allergy and Infectious Diseases, National Institutes of Health, Bethesda, Maryland, USA; University of Pittsburgh School of Medicine

**Keywords:** CKII, E3 ligase, NF-kB, cullin ring ligase, innate immunity, rotavirus

## Abstract

The rotavirus nonstructural protein NSP1 repurposes cullin-RING E3 ubiquitin ligases (CRLs) to antagonize innate immune responses. By functioning as substrate adaptors of hijacked CRLs, NSP1 causes ubiquitination and proteasomal degradation of host proteins that are essential for expression of interferon (IFN) and IFN-stimulated gene products. The target of most human and porcine rotaviruses is the β-transducin repeat-containing protein (β-TrCP), a regulator of NF-κB activation. β-TrCP recognizes a phosphorylated degron (DSGΦXS) present in the inhibitor of NF-κB (IκB); phosphorylation of the IκB degron is mediated by IκB kinase (IKK). Because NSP1 contains a C-terminal IκB-like degron (ILD; DSGXS) that recruits β-TrCP, we investigated whether the NSP1 ILD is similarly activated by phosphorylation and whether this modification is required to trigger the incorporation of NSP1 into CRLs. Based on mutagenesis and phosphatase treatment studies, we found that both serine residues of the NSP1 ILD are phosphorylated, a pattern mimicking phosphorylation of IκB. A three-pronged approach using small-molecule inhibitors, small interfering RNAs, and mutagenesis demonstrated that NSP1 phosphorylation is mediated by the constitutively active casein kinase II (CKII), rather than IKK. In coimmunoprecipitation assays, we found that this modification was essential for NSP1 recruitment of β-TrCP and induced changes involving the NSP1 N-terminal RING motif that allowed formation of Cul3-NSP1 complexes. Taken together, our results indicate a highly regulated stepwise process in the formation of NSP1-Cul3 CRLs that is initiated by CKII phosphorylation of NSP1, followed by NSP1 recruitment of β-TrCP and ending with incorporation of the NSP1–β-TrCP complex into the CRL via interactions dependent on the highly conserved NSP1 RING motif.

## IMPORTANCE

Rotavirus is a segmented double-stranded RNA virus that causes severe diarrhea in young children. A primary mechanism used by the virus to inhibit host innate immune responses is to hijack cellular cullin-RING E3 ubiquitin ligases (CRLs) and redirect their targeting activity to the degradation of cellular proteins crucial for interferon expression. This task is accomplished through the rotavirus nonstructural protein NSP1, which incorporates itself into a CRL and serves as a substrate recognition subunit. The substrate recognized by the NSP1 of many human and porcine rotaviruses is β-TrCP, a protein that regulates the transcription factor NF-κB. In this study, we show that formation of NSP1 CRLs is a highly regulated stepwise process initiated by CKII phosphorylation of the β-TrCP recognition motif in NSP1. This modification triggers recruitment of the β-TrCP substrate and induces subsequent changes in a highly conserved NSP1 RING domain that allow anchoring of the NSP1–β-TrCP complex to a cullin scaffold.

## INTRODUCTION

**FIG 1:**
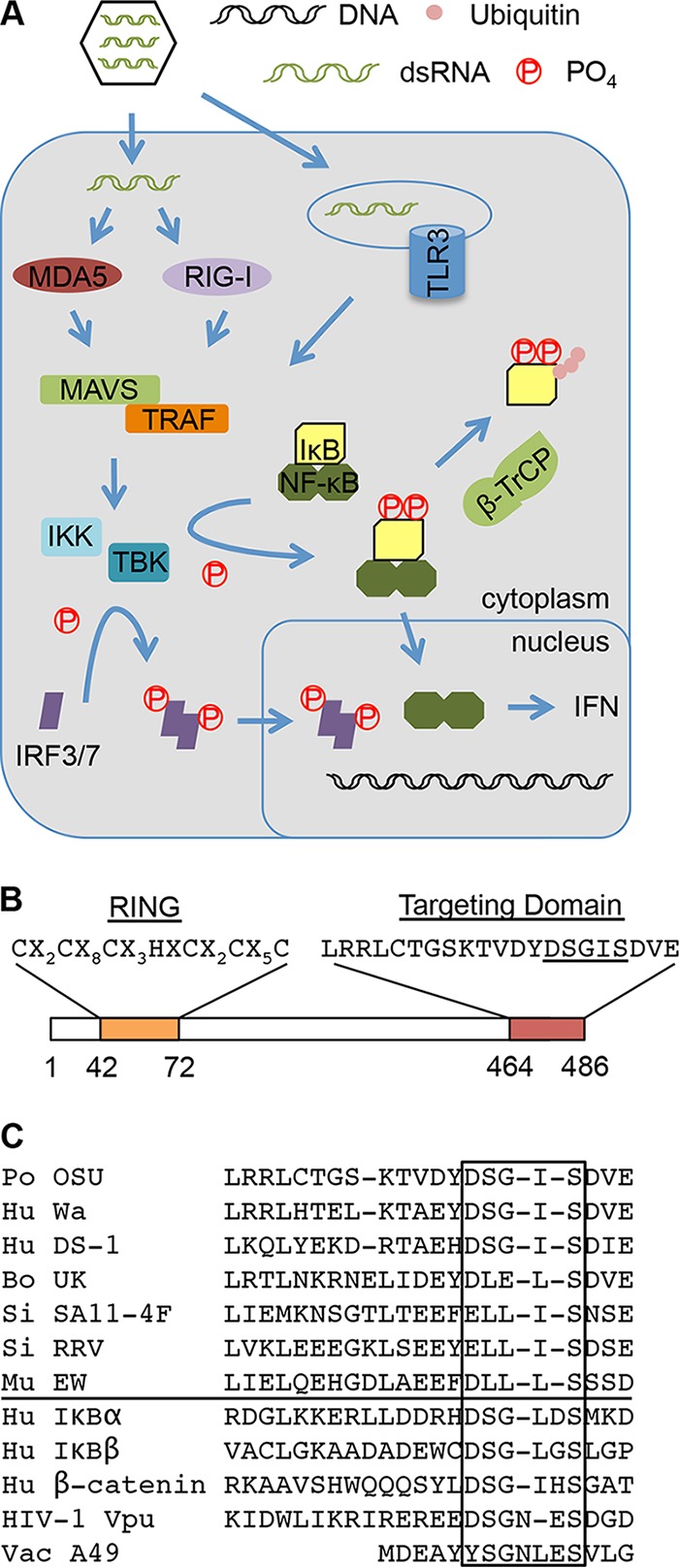
Targeting of the interferon activation pathway by rotavirus NSP1. (A) Recognition of viral double-stranded RNA causes host RNA sensors RIG-I, MDA5, and/or Toll-like receptor 3 (TLR3) to interact with MAVS and TRAF adaptor proteins, activating the kinases IKK and TBK1. IKK directs the phosphorylation of serine residues in the DSGΦXS motif of IκB, generating a phosphodegron that is recognized by β-TrCP, a substrate adaptor of an E3 cullin-RING ligase. β-TrCP mediates the ubiquitination and degradation of IκB, freeing the NF-κB subunits to translocate to the nucleus and promote IFN expression. TBK1 directs phosphorylation of IRF3/7, inducing their dimerization and nuclear translocation and further stimulating IFN expression. (B) Key features of NSP1 proteins include a conserved N-terminal RING-like domain and a variable C-terminal substrate targeting domain. The C-terminal targeting domain of OSU NSP1 includes an ILD motif (underlined). (C, above line) Alignment revealing the presence of ILDs in the NSP1 proteins of common porcine (OSU) and human (Wa and DS1) rotavirus strains but their absence in the NSP1 proteins of bovine (UK), simian (SA11-4F, RRV), and murine (EW) virus strains. (Below line) IκB-like degron sequences within other known binding partners of β-TrCP, including the HIV Vpu and vaccinia virus A49 proteins. Regions that include IκB degron and degron-like sequences are boxed.

Rotaviruses, members of the family *Reoviridae*, are a leading cause of severe life-threatening diarrhea in infants and children under 5 years of age (1, 2). The segmented double-stranded RNA of these viruses encode six structural proteins (VP1 to VP4, VP6, and VP7) and six nonstructural proteins (NSP1 to NSP6) (2). Rotaviruses antagonize the expression of interferon (IFN) and IFN-stimulated gene (ISG) products, chiefly through the action of NSP1, the sole protein product of viral genome segment 5 (3). NSP1 is the least conserved of the 12 rotavirus proteins, including VP4 and VP7, the outer capsid proteins of the virus that are under immunological pressure (4). However, the NSP1 proteins of group A rotaviruses (our subject here) have a similar predicted secondary structure and share the same N-terminal RING domain motif (4). The C-termini of NSP1 proteins have variable C-terminal substrate recognition domains that recruit and induce the proteasomal degradation of host proteins involved in IFN and ISG expression (4, 5). The NSP1 C-terminal domain of most human and porcine rotaviruses targets the β-transducin repeat-containing protein (β-TrCP) for degradation (6), a protein critical for activation of the transcription factor nuclear factor-κB (NF-κB) (Fig. 1A). In contrast, the targets of many animal rotaviruses, including simian, bovine, equine, and murine strains, are the IFN regulatory factors (e.g., IRF3 and IRF7) (1, 4, 5).

Human and porcine NSP1 proteins recognize β-TrCP through the presence of a DSGXS motif in their C-terminal substrate recognition domain (Fig. 1B and C). This motif mimics the DSGΦXS degron present in inhibitor of NF-κB (IκB) (5). When bound to NF-κB, IκB prevents movement of the NF-κB p65-p50 heterodimer from the cytoplasm to the nucleus, where the transcription factor stimulates IFN and ISG promoter activity (Fig. 1A) (7). Induction of upstream innate immune signaling cascades by viruses and other pathogens triggers phosphorylation of the IκB degron by the IκB kinase (IKK) complex (8). Recognition of IκB’s phosphorylated degron (phosphodegron) by β-TrCP, a substrate adaptor of an E3 ubiquitin ligase, triggers IκB ubiquitination and proteasomal degradation, allowing nuclear translocation of the NF-κB heterodimer (8). By recruiting β-TrCP to its C-terminal IκB-like degron (ILD), NSP1 is able to interfere with the interaction of β-TrCP with phosphorylated IκB, thus thwarting β-TrCP-mediated degradation of IκB and NF-κB activation. Indeed, mutagenesis and deletion analyses have indicated that the NSP1 C-terminal ILD has an essential role in the formation of NSP1–β-TrCP complexes and in the degradation of β-TrCP in infected cells (5).

Recent studies by Lutz et al. (9) and Ding et al. (10) suggested that NSP1 induces β-TrCP degradation by functioning as a substrate adaptor subunit of hijacked E3 cullin-RING ligases (CRLs). CRLs are large multicomponent assemblages that coordinate the activities of many cellular processes, including innate immune responses to viral infection (11), by facilitating directed degradation of substrates. Central to CRL function, a cullin scaffold protein bridges ubiquitin-charged E2 conjugating enzymes to substrates anchored to substrate adaptor proteins. Modification of the cullin component by neddylation activates the CRL, resulting in the transfer of ubiquitin from E2 onto the substrate (11, 12). NSP1 is believed to be a component of a cullin-3 CRL (Cul3 CRL) with specificity for β-TrCP. In contrast, the E3 ligase responsible for targeting IκB for proteasomal degradation is assembled on a cullin-1 (Cul1) scaffold that anchors a substrate recognition complex consisting of Skp1 and the F-box adaptor protein β-TrCP; this E3 is termed the Skp1–Cul1–F-box (SCF^β-TrCP^) CRL (7, 13).

Because of its critical role in innate immune responses, the function of the SCF^β-TrCP^ CRL and its components are primary targets of many viral proteins, in addition to those of rotavirus (14). For example, through its interaction with β-TrCP, HIV Vpu redirects the activity of the SCF^β-TrCP^ CRL, causing the ubiquitination and degradation of CD4 (15, 16). Similarly, by sequestering β-TrCP, the vaccinia virus A49 protein prevents the SCF^β-TrCP^ CRL from targeting IκB for degradation (17). Unlike NSP1, A49 does not function as part of an E3 ligase and is not associated with β-TrCP degradation.

Phosphorylation of the IκB degron by the IKK kinase complex triggers a sequence of events that lead to the assembly of the SCF^β-TrCP^ CRL, which induces IκB degradation and promotes NF-κB activation (18). Whether rotavirus NSP1 relies on a similar pathway to assemble Cul3 CRLs that target β-TrCP for degradation is unknown. In this study, we determined that the NSP1 ILD is phosphorylated and that the kinase responsible for this modification is the constitutively active casein kinase II (CKII), rather than the kinase that phosphorylates the IκB degron (IKK). Moreover, we found that phosphorylation of the NSP1 ILD not only enables formation of a stable NSP1–β-TrCP complex but also triggers changes involving the NSP1 N-terminal RING domain that result in Cul3 interactions. Thus, CKII phosphorylation is a critical initiating event not only in the ability of NSP1 to recruit β-TrCP but also in its ability to incorporate itself into a CRL that induces the ubiquitination and degradation of β-TrCP.

## RESULTS

### The NSP1 ILD is phosphorylated.

**FIG 2:**
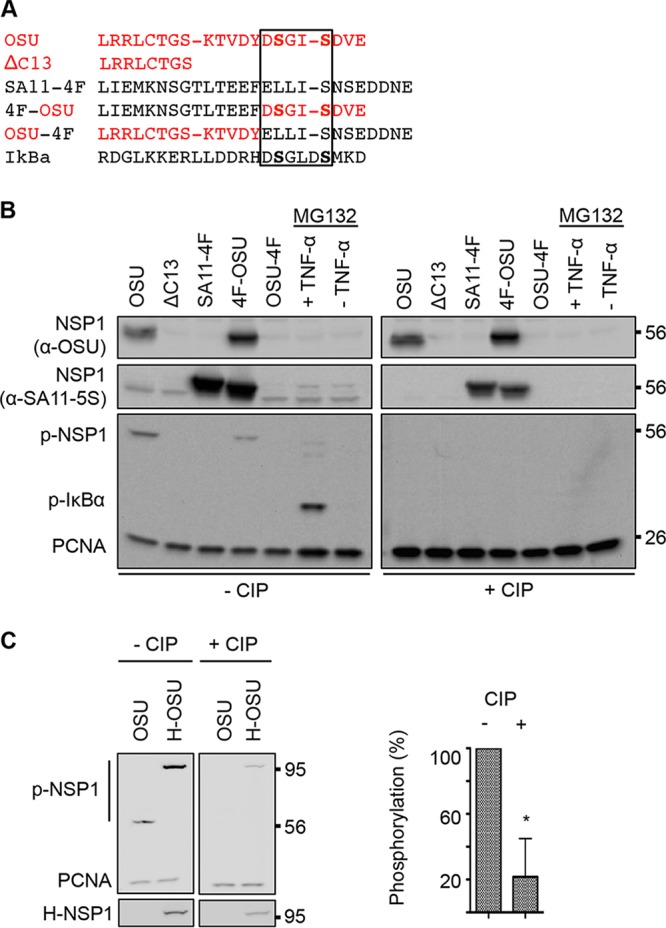
Phosphorylation of transiently expressed OSU NSP1. (A) Alignment of C-terminal NSP1 sequences and a portion of IκB containing the DSGΦXS phosphodegron. Red lettering indicates OSU-specific residues. The phosphodegron region is boxed. (B) HEK293T cells were transfected with NSP1 expression vectors or maintained in the presence of MG132 with or without TNF-α, beginning at 20 h p.t. Lysates prepared from cells at 24 h p.t. were resolved by gel electrophoresis, in duplicate, and blotted onto nitrocellulose membranes. Membranes were mock treated (-CIP) or treated with CIP in parallel. Mock- and CIP-treated membranes were probed with PCNA antibody, p-IκB antibody to detect phosphorylated NSP1 (p-NSP1) and IκB (p-IκB), OSU NSP1 antibody to detect OSU and 4F-OSU NSP1, and SA11-5S antibody to detect SA11-4F and 4F-OSU NSP1. (C) Lysates prepared from HEK293T cells transfected with expression vectors for OSU NSP1 (OSU) or HALO-tagged OSU NSP1 (H-OSU) were resolved by gel electrophoresis and blotted onto nitrocellulose membranes, in duplicate. Mock- and CIP-treated membranes were probed with p-IκB antibody to detect p-NSP1 and with antibodies to the HALO tag and PCNA. Levels of phosphorylated H-OSU were calculated relative to PCNA levels and normalized to 100% for the untreated sample. Data shown are from two independent experiments (means ± standard deviations). *, *P* < 0.05.

The interaction of β-TrCP with IκB requires phosphorylation of the two serine residues in the IκB degron, DSGΦXS (8). By extrapolation, interaction of β-TrCP with NSP1 may similarly require phosphorylation of the two serine residues in the NSP1 C-terminal ILD, DSGXS. To determine whether NSP1 is phosphorylated, we transiently expressed various untagged forms of NSP1 in HEK293T cells, including wild-type OSU NSP1 (WT) and a C-truncated form of OSU NSP1 lacking the ILD (ΔC13) (Fig. 2A and B). Also expressed were two chimeras produced by combining portions of the OSU and SA11-4F NSP1 proteins. The OSU-4F chimera is identical in sequence to OSU NSP1 except that its last 8 residues have been replaced with the corresponding region of SA11-4F NSP1. Thus, the OSU-4F NSP1 chimera lacks an ILD. In contrast, the 4F-OSU chimera is identical in sequence to SA11-4F NSP1 except that its last 12 residues have been replaced with the corresponding region of OSU NSP1. Thus, the 4F-OSU NSP1 chimera contains an ILD. The phosphorylation status of transiently expressed WT and ΔC13 NSP1 and OSU-4F and 4F-OSU chimeras was examined in an immunoblot assay using a monoclonal antibody (p-IκB) that specifically recognized the fully phosphorylated form of the IκB degron (DpSGΦXpS). The assay showed that only those NSP1 proteins containing an ILD (WT, 4F-OSU) were recognized by p-IκB antibody (Fig. 2B). These results suggested that OSU NSP1 is a phosphoprotein and that its ILD undergoes phosphorylation.

**FIG 3:**
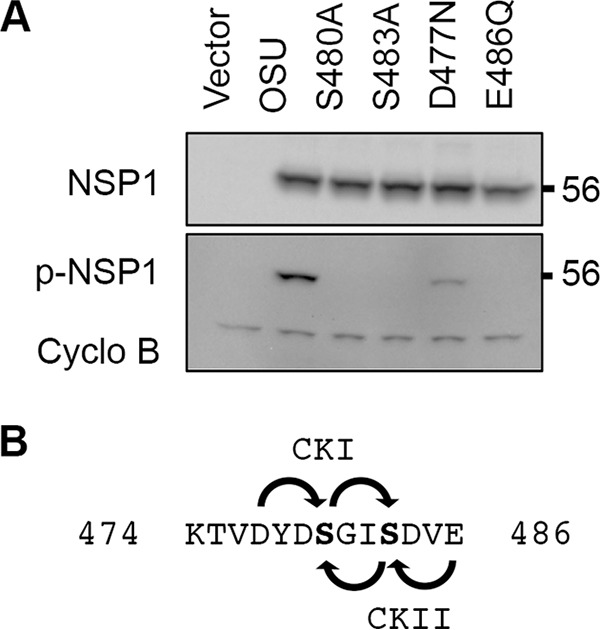
Effect of priming loop mutations on NSP1 phosphorylation. (A) Lysates prepared from HEK293T cells expressing WT OSU NSP1 and forms of the protein with the indicated mutations were analyzed by immunoblot assay using p-IκB antibody to recognize p-NSP1, OSU NSP1 antibody, and PCNA antibody. (B) Predicted phosphorylation patterns of the OSU NSP1 ILD by CKI and CKII, using D477 and E486 as priming residues, respectively.

To further evaluate the possibility that the ILD of OSU NSP1 was phosphorylated, blots of transiently expressed WT and ΔC13 NSP1 and OSU-4F and 4F-OSU chimeras were incubated with calf intestinal alkaline phosphatase (CIP) prior to probing with p-IκB antibody. The analysis demonstrated that CIP pretreatment reduced recognition of WT and 4F-OSU NSP1 by p-IκB antibody (Fig. 2B). Similar results were obtained when we utilized HALO-tagged OSU NSP1 (H-NSP1) in place of untagged NSP1 (Fig. 2C). CIP pretreatment reduced recognition of H-NSP1 by p-IκB antibody, even though the protein remained present, as determined using an anti-HALO antibody. Likewise, in control experiments, CIP treatment reduced recognition of p-IκB formed in cells treated with tumor necrosis factor alpha (TNF-α), a cytokine that upregulates IKK and activates NF-κB (Fig. 2B). The importance of the ILD in recognition of NSP1 by p-IκB antibody was also investigated via a mutagenesis study. This analysis showed that replacement of serine residues with alanine in the IκB-like DSGXS motif, either individually or in combination, prevented p-IκB antibody from recognizing OSU NSP1 (Fig. 3A). Together, these findings indicate that the ILD of OSU NSP1 is phosphorylated in a manner that mimics the IκB degron, with both serine resides of the DSGXS motif phosphorylated.

### NSP1 encoded by various rotavirus strains is phosphorylated.

**FIG 4:**
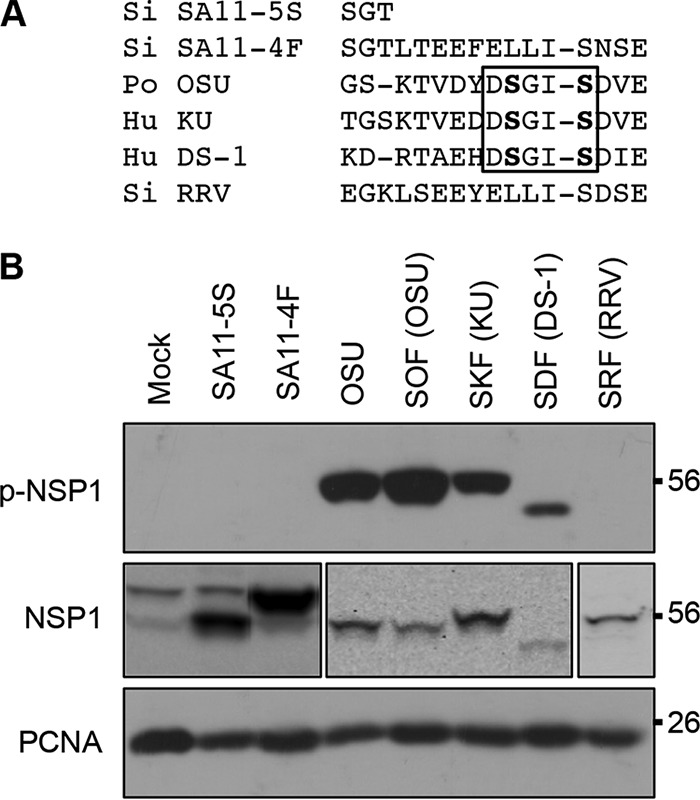
Phosphorylation of NSP1 in rotavirus-infected cells. (A) Alignment of C-terminal sequences of NSP1 proteins, with the ILD boxed. (B) HT29 cells were infected with SA11-4F, SA11-5S, or OSU virus strains or monoreassortant SA11-L2 virus strains expressing OSU (SOF), KU (SKF), DS-1 (SDF), or RRV (SRF) NSP1. SA11-5S expresses a mutant form of NSP1 that lacks the 13 terminal residues of wild type SA11-4F NSP1. Lysates prepared from the infected cells at 15 h p.i. were analyzed by immunoblot assay using p-IκB antibody to detect p-NSP1 and PCNA antibody. To detect total NSP1, lysates from mock-, SA11-4F-, and SA11-5S-infected cells were probed with antibody against SA11-5S NSP1, lysates from OSU-, SOF-, SKF-, and SDF-infected cells were probed with antibody against OSU NSP1, and the lysate from RRV-infected cells was probed with antibody against RRV NSP1 (4).

To assess whether NSP1 proteins expressed during infection were phosphorylated, human HT29 cells were infected with rotavirus strains OSU, SA11-4F, and SA11-5S and with a panel of monoreassortant rotaviruses containing various segment 5 RNAs in an SA11-L2 background. The reassortant viruses included SOF, SKF, SDF, and SRF, which express the NSP1 proteins of porcine OSU, human KU, human DS-1, and simian RRV strains, respectively. Of these NSP1 proteins, only those encoded by OSU, KU, and DS1 viruses contained an ILD (Fig. 4A). Rotavirus-infected HT29 cells were harvested at 10 h postinfection (p.i.), and proteins in HT29 lysates were analyzed by immunoblot assay using p-IκB antibody. The results showed that those NSP1 proteins containing an ILD were recognized by p-IκB antibody (Fig. 4B). Thus, NSP1 proteins with ILDs undergo phosphorylation in rotavirus-infected cells, and this protein modification occurs for rotavirus strains isolated from a variety of animal species (e.g., human, simian, porcine). OSU, KU, and DS1 NSP1 proteins produced in rotavirus-infected simian MA104 and porcine PK15 cells were similarly recognized by p-IκB antibody, suggesting that phosphorylation of the ILD takes place regardless of the species origin of the host cell line (data not shown). While our results showed that SA11-4F and RRV NSP1 proteins were not recognized by the p-IκB antibody, these data do not exclude the possibility that these proteins are phosphorylated at sites other than an ILD motif.

### NSP1 is phosphorylated by CKII.

Interaction of β-TrCP with IκB is dependent on phosphorylation of the IκB degron by the IKKβ subunit (19). To determine whether the NSP1 ILD motif was phosphorylated by the same kinase, or a different one, the Eukaryotic Linear Motif resource (elm.eu.org) (20) was used to identify potential kinase recognition motifs in NSP1 proteins. The analysis indicated that NSP1 proteins with an ILD lack a recognition site for IKKβ kinase. Instead, the NSP1 ILD was found to be situated within recognition motifs for CKI and CKII (Fig. 3B) (5). CKI and CKII direct phosphorylation after priming at a negatively charged amino acid residue (e.g., aspartic acid, glutamic acid) located three positions upstream or downstream of a target serine residue, respectively (21–23). Examination of NSP1 proteins indicated that such amino acids are present at the putative priming positions for CKI and CKII in the NSP1 ILD motif [(*D/E*)YD**S**GI**S**DV*E*] (the priming positions are italicized, and phosphoserines appear in bold). Whether CKI or CKII was involved in NSP1 phosphorylation was examined by transient expression of OSU NSP1 proteins in which the putative priming residues had been mutated. The results showed that a Glu-to-Gln mutation of the putative CKII priming residue prevented recognition of OSU NSP1 by p-IκB antibody, whereas an Asp-to-Asn mutation of the putative CKI priming residue did not (Fig. 3A). These mutagenesis data indicate that CKII is responsible for phosphorylation of the NSP1 ILD.

**FIG 5:**
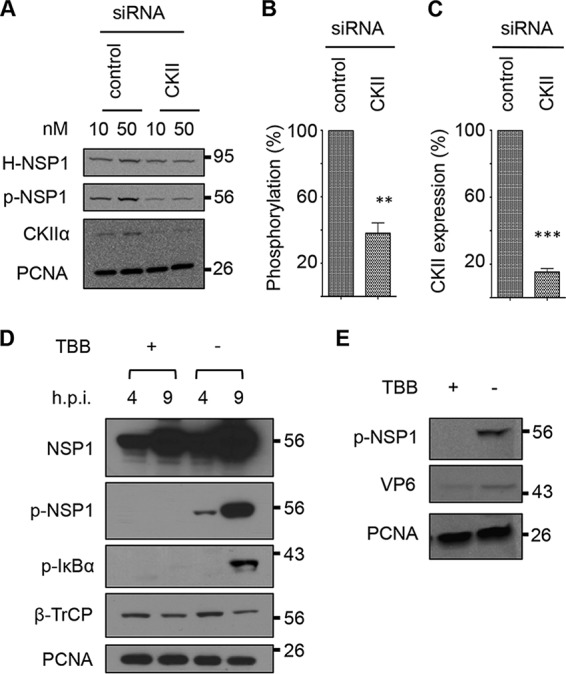
Effect of CKII inhibition on NSP1 phosphorylation. (A) HEK293T cells were cotransfected with a vector expressing HALO-tagged NSP1 (H-NSP1) and a 10 or 50 μM siRNA pool targeting the CKII RNA or representing a scrambled siRNA control pool. At 48 h p.t., cells were collected and protein samples were analyzed by immunoblot assay with antibodies specific for the HALO tag, p-NSP1, CKIIα, and PCNA. Band intensities from two independent experiments using 50 mM concentration of siRNA pools were determined with an Azure digital imager. (B) Levels of OSU NSP1 phosphorylation were calculated by dividing p-NSP1 intensity values by those for H-NSP1 and normalizing the results to 100% for the control siRNA sample. (C) CKII levels were calculated by dividing CKII intensity values by those for PCNA and normalizing the results to 100% for the control siRNA sample. (D) HT29 cells were infected with OSU at an MOI of 5 and treated with TBB or DMSO at 2 h p.i. At 4 and 9 h p.i., cells were collected and lysed, and proteins in the samples were resolved by electrophoresis and examined by immunoblot assay with antibodies recognizing OSU NSP1, p-NSP1, p-IκB, β-TrCP, or PCNA. (E) PK15 cells were infected with OSU at an MOI of 5 and mock treated or treated with TBB. Cells were harvest at 9 h p.i., and proteins in the samples were examined by immunoblot assay with antibodies to p-NSP1, rotavirus VP6, and PCNA.

To further evaluate the importance of CKII activity on NSP1 phosphorylation, HEK293T cells were transfected with a vector expressing HALO-tagged OSU NSP1 and either a small interfering RNA (siRNA) specific for CKII RNA or a scrambled control siRNA pool (Fig. 5A to C). Immunoblot analysis showed that treatment with CKII siRNA significantly reduced levels of CKII expression and phosphorylated NSP1 (Fig. 5B and C) relative to levels after treatment with scrambled siRNAs, supporting the hypothesis that CKII mediates phosphorylation of the NSP1 ILD. The contribution of CKII to NSP1 phosphorylation was also examined by treating OSU-infected human HT29 and porcine PK15 cells with 4,5,6,7-tetrabromobenzotriazole (TBB), a highly specific cell-permeable nucleoside inhibitor of CKII (24). Whole-cell lysates prepared from the infected cells at 4 and/or 9 h p.i. were analyzed by immunoblot analysis with a cross-reacting (SA11-5S) antibody that recognizes total OSU NSP1 (4) and the p-IκB antibody to detect phosphorylated NSP1 and IκB (Fig. 5D and E). The results showed that TBB inhibited the accumulation of phosphorylated NSP1 in both HT29 and PK15 infected cells. The detection of NSP1 in TBB-treated cells with the cross-reacting (SA11-5S) antibody ruled out the possibility that the inhibitor prevented NSP1 expression. Taken together, the results obtained using CKII siRNAs and TBB demonstrated that CKII is responsible for phosphorylation of the NSP1 ILD.

### NSP1 phosphorylation is required for NSP1–β-TrCP interactions.

**FIG 6:**
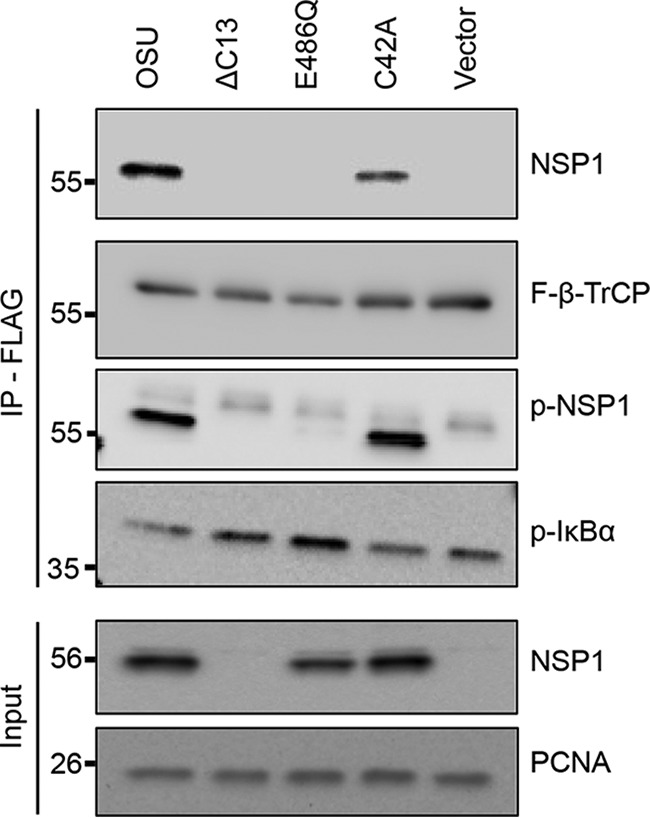
Effect of NSP1 phosphorylation on interactions with β-TrCP. HEK293T cells were transfected with vectors expressing FLAG–β-TrCP and WT or mutant OSU NSP1. Lysates prepared from the cells at 24 h p.t. were incubated with anti-FLAG resin to immunoprecipitate complexes containing FLAG–β-TrCP. Input fractions and eluted proteins were analyzed by immunoblot assay with antibodies specific for NSP1, FLAG, p-NSP1, p-IκB, and PCNA. The OSU NSP1 antibody was generated using a peptide representing the C terminus of the OSU NSP1 proteins (4). As a result, the OSU NSP1 antibody was not able to recognize the product of the OSU ΔC13 expression vector (5).

Because IκB must undergo phosphorylation for interaction with β-TrCP (8), we predicted that NSP1 may likewise require phosphorylation for interaction with β-TrCP. To examine this possibility, FLAG-tagged β-TrCP (F-β-TrCP) was transiently expressed in HEK293T cells with WT NSP1 or mutant forms of NSP1 that lacked either an ILD (ΔC13) or the CKII priming residue (E486Q), or that contained a mutation of a conserved cysteine residue in the N-terminal RING domain (C42A). F-β-TrCP was recovered from lysates prepared from the transfected cells by affinity purification with anti-FLAG resin, and proteins that copurified with β-TrCP were identified in an immunoblot assay. The results indicated that WT NSP1 and NSP1 with a mutated RING domain (C42A) interacted with F–β-TrCP (Fig. 6). In contrast, NSP1 that lacked an ILD motif (ΔC13) or that was unable to undergo CKII phosphorylation (E486Q) did not form complexes with F-β-TrCP (Fig. 6). These data indicate that NSP1 must contain a phosphorylated ILD for stable interaction with β-TrCP. Interestingly, the data also suggest that the NSP1 RING domain may not have a role in modulating NSP1–β-TrCP interactions, since the NSP1 RING mutant C42A retained binding activity for β-TrCP.

### NSP1 phosphorylation is required for incorporation into CRLs.

**FIG 7:**
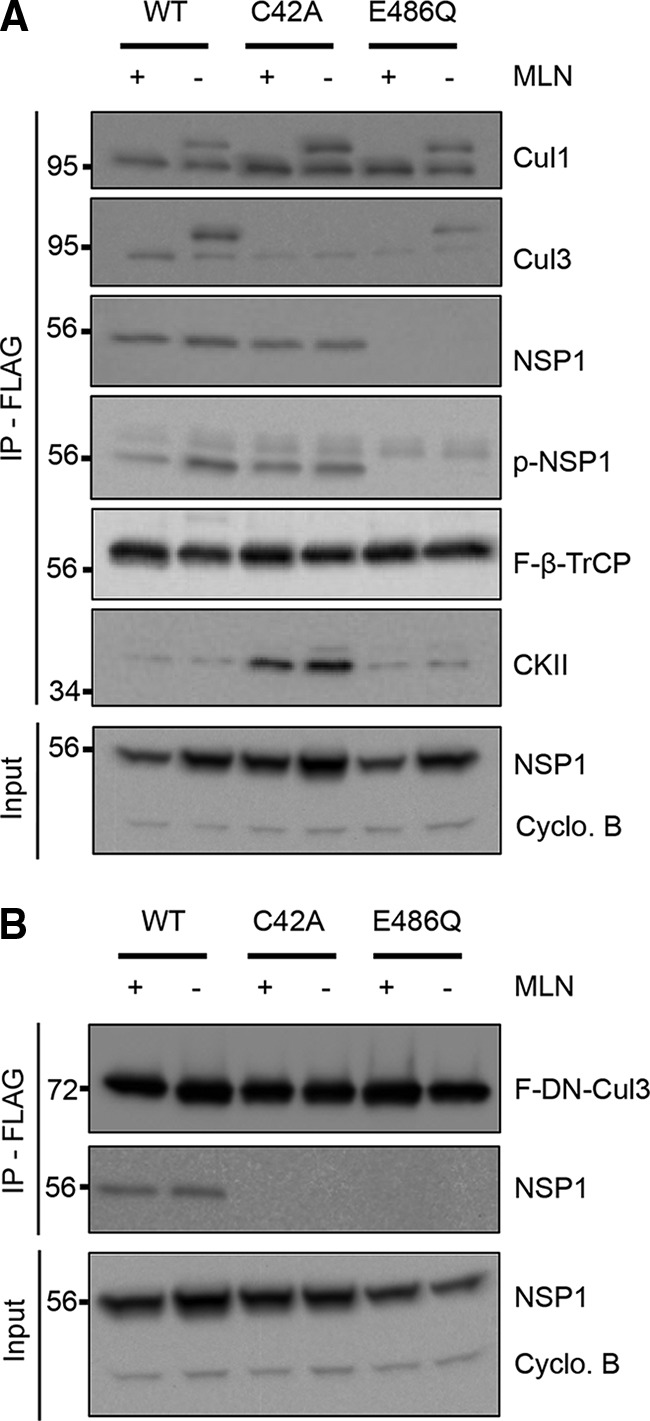
Effect of NSP1 phosphorylation on interactions with Cul3. HEK293T cells were transfected with vectors expressing WT or mutant C42A or E486Q OSU NSP1 and FLAG–β-TrCP (A) or FLAG (F)-DN-Cul3 (B). Transfected cells were mock treated or treated with the neddylation inhibitor MLN4964 (MLN). Lysates prepared from the cells at 24 h p.t. were incubated with anti-FLAG resin to immunoprecipitate complexes containing FLAG-tagged proteins. Input fractions and eluted proteins were analyzed by immunoblot assay with antibodies specific for Cul1, Cul3, NSP1, p-NSP1, FLAG, CKIIα, and cyclophilin B.

Previous reports indicated that NSP1 functions as a substrate adaptor of Cul3 CRLs (9, 10). To address whether incorporation of NSP1 into Cul3 CRLs is influenced by the phosphorylation status of the NSP1 ILD, we transiently expressed F-β-TrCP with WT NSP1 or mutant NSP1 proteins C42A or E486Q in HEK239T cells. We stabilized CRLs that formed in transfected cells by the addition of MLN4924, a NEDD8-activating enzyme inhibitor that inhibits cullin neddylation and CRL disassociation (25). Immunoprecipitates containing F-β-TrCP were recovered from transfected cell lysates by using an anti-FLAG affinity resin (Fig. 7A). Characterization of the immunoprecipitates indicated that F-β-TrCP interacted with neddylated and nonneddylated forms of Cul1, consistent with previous reports showing that β-TrCP is a component of the SCF^β-TrCP^ E3 ligase (Fig. 7A). The analysis also indicated that F-β-TrCP interacted with WT and C42A p-NSP1, but not with E486Q NSP1 (Fig. 7A). This finding argues that the interaction of β-TrCP with NSP1 is dependent on phosphorylation of the NSP1 ILD, consistent with data presented in Fig. 6. Surprisingly, analysis of F-β-TrCP immunoprecipitates recovered from cells expressing C42A NSP1 indicated that C42A NSP1-β-TrCP complexes were formed that contained CKII, the kinase responsible for phosphorylation of the NSP1 ILD (Fig. 7A). In contrast, relatively little CKII was detected in F-β-TrCP immunoprecipates recovered from cells in which F-β-TrCP was coexpressed with WT NSP1 or a mutant form of NSP1 that cannot undergo phosphorylation (E486Q). These data suggest that CKII has affinity for NSP1 and that the interaction between the two proteins requires the CKII E486 priming residue and/or phosphorylation of the NSP1 ILD. Moreover, these data suggest that CKII interaction with the NSP1–β-TrCP complex does not require a WT RING domain in the NSP1 protein.

More Cul3 appeared to be present in F-β-TrCP immunoprecipitates recovered from transfected cells in which F-β-TrCP was coexpressed with WT NSP1 than when coexpressed with C42A or E486Q NSP1 (Fig. 7A). To further evaluate this possibility, we transiently expressed FLAG-tagged dominant-negative Cul3 [F-(DN)Cul3] with WT, C42A, or E486Q NSP1 in HEK293T cells (Fig. 7B). (DN)Cul3 is a carboxyl truncated form of Cul3 that forms stable complexes with F-box adaptor proteins and substrates but is unable to interact with Rbx1 and the E2 ubiquitin-conjugating enzyme (26). Immunoblot analysis of F-(DN)Cul3 immunoprecipitates indicated that WT NSP1, but neither NSP1 with a mutated RING motif (C42A) nor a phosphorylation-defective ILD motif (E486Q), interacted with Cul3 (Fig. 7B). These results suggest that the NSP1 RING domain plays an important role in NSP1-Cul3 interactions and, as a consequence, the incorporation of NSP1 into Cul3 CRLs. However, the fact that E486Q NSP1 did not bind Cul3, despite the presence of a WT RING domain, argues that the CKII E486 priming residue and/or phosphorylation of the NSP1 ILD motif precedes the activity of the RING domain in promoting NSP1-Cul3 interactions. Taken together, these data suggest an ordered association of NSP1 with CRLs and its protein target; NSP1 interactions with CKII lead to phosphorylation of its C-terminal ILD and binding to β-TrCP targets, followed by CKII release and Cul3 binding.

### Relationship between NSP1 phosphorylation and β-TrCP degradation.

**FIG 8:**
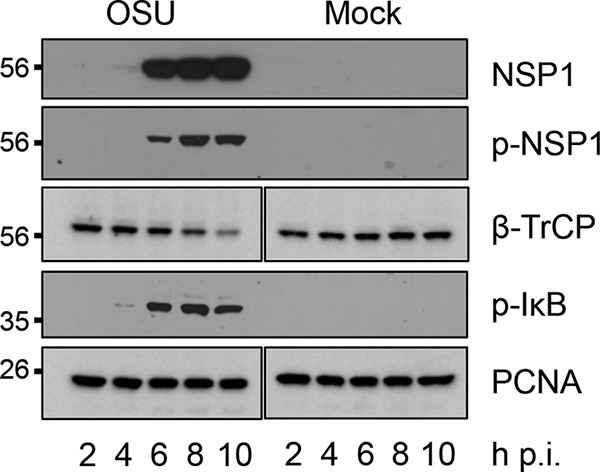
Accumulation of p-NSP1, β-TrCP, and p-IκB during viral infection. HT29 cells were mock infected or infected with OSU rotavirus (MOI of 5) and harvested from 2 to 10 h p.i. Cellular lysates were resolved by gel electrophoresis and transferred onto nitrocellulose membranes. Blots were probed with antibodies specific for NSP1, p-NSP1, β-TrCP, p-IκB, and PCNA.

The results presented above indicate that the interaction of phosphorylated NSP1 with β-TrCP is not dependent on the incorporation of NSP1 into a CRL, raising the question of whether NSP1 could inhibit NF-κB-activation by simply sequestering β-TrCP away from its IκB target. To examine this possibility, HT29 cells were mock infected or infected with OSU rotavirus. Lysates prepared from the cells at 2, 4, 6, 8, and 10 h p.i. were analyzed in an immunoblot assay with antibodies recognizing phosphorylated NSP1 and IκB, and β-TrCP (Fig. 8). The analysis showed that NSP1 was initially detected by 4 h p.i., with levels sharply increasing by 6 h p.i. Phosphorylated NSP1 was first observed by 6 h p.i., with levels further increasing until 8 h p.i. Phosphorylated IκB was initially detected by 4 h p.i., indicating activation of the IKK signaling cascade. The level of phosphorylated IκB markedly increased by 6 h p.i. and remained high through at least 10 h p.i., suggesting a failure of β-TrCP to efficiently recruit and induce the degradation of IκB in infected cells. Notably, degradation of β-TrCP was not apparent until after 6 to 8 h p.i., well after significant accumulation of phosphorylated IκB was observed. Thus, even though β-TrCP was present in infected cells at 6 to 8 h p.i., its presence was not correlated with the degradation of phosphorylated IκB. These results raise the likelihood that β-TrCP degradation may not be the only mechanism used by NSP1 to inhibit IκB degradation and NF-κB activation. Instead, it is possible that NSP1 can accomplish these tasks simply by binding β-TrCP, thereby interfering with the ability of NF-κB to interact with its IκB target.

## DISCUSSION

**FIG 9:**
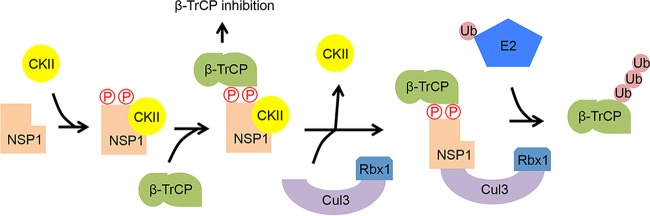
Predicted steps in the CKII-dependent incorporation of NSP1 into a Cul3 CRL. The constitutively active kinase CKII interacts with NSP1, leading to phosphorylation of the NSP1 ILD. The affinity of β-TrCP for the phosphorylated degron results in the formation of an NSP1–β-TrCP–CKII complex. CKII is released as the NSP1–β-TrCP complex interacts with the CRL assembly intermediate Cul3-Rbx1. Recruitment of a charged E2 conjugating enzyme to Rbx1 results in the formation of a Cul3 CRL that causes the ubiquitination and proteasomal degradation of β-TrCP.

In this study, we determined that rotavirus NSP1 is phosphorylated and that this modification represents a critical initiating event in the interaction of NSP1 with β-TrCP and in the incorporation of NSP1 into Cul3 CRLs (Fig. 9). Our analysis indicated that both serine residues in the OSU NSP1 ILD (DSGXS) undergo phosphorylation, mimicking phosphorylation of the two conserved serine residues of the IκB degron. The NSP1 proteins of all tested rotaviruses containing an ILD were phosphorylated, regardless of the species origin of the virus or the species origin of the cell line used to grow the virus. The phosphorylated NSP1 proteins included those belonging to genotypes A1 and A2, representing the types of NSP1 genes found in nearly all rotaviruses that cause disease in humans (3). NSP1 proteins with ILD motifs, whether transiently expressed in cells transfected with cytomegalovirus (CMV) transcription vectors or expressed in rotavirus-infected cells, were phosphorylated. There was no evidence suggesting that other rotavirus gene products were necessary or affected NSP1 phosphorylation. Experiments performed with the NSP1 C42A mutant indicated that the protein’s N-terminal RING region does not have a role in regulating ILD phosphorylation.

Phosphorylation assays carried out with small-molecule inhibitors, siRNAs, and mutagenized expression vectors all indicated that CKII, a ubiquitous constitutively active serine-threonine kinase (27), is responsible for phosphorylation of the NSP1 ILD. Based on the time course experiment results presented in Fig. 8, phosphorylated NSP1 was detected in OSU-infected cells at approximately the same time (6 h p.i.) that NSP1 began to accumulate (Fig. 8). Thus, NSP1 is likely primed for interaction with β-TrCP soon after synthesis, in contrast to IκB, which is dependent on activation of the upstream IKK signaling cascade to undergo phosphorylation (7). Indeed, by utilizing CKII, NSP1 can potentially undergo phosphorylation and target β-TrCP for degradation before pattern recognition receptors of infected cells trigger innate immune signaling pathways and IκB phosphorylation. Our coimmunoprecipitation studies suggested that CKII not only phosphorylates the NSP1 ILD but also transiently binds to NSP1 at a step that occurs prior to the incorporation of NSP1 into CRLs. Whether the entire CKII tetrameric complex (28) is bound to NSP1, or just the catalytic subunit recognized by the CKII antibody, is not known. The source of the CKII kinase activity may be the COP9 signalsome (29), a multisubunit protein complex responsible for deneddylating cullins and regulating CRL activity (30). Notably, NSP1 is not the only rotavirus protein known to be phosphorylated by a cellular kinase during virus replication. In particular, one of the major building blocks of rotavirus viroplasms (NSP5) undergoes extensive phosphorylation by CKI (31).

Our coimmunoprecipitation experiment results suggest that the CKII–NSP1–β-TrCP complex is a precursor of the Cul3–NSP1–β-TrCP complex, opening the possibility that interaction of Cul3 with NSP1 displaces CKII. Interestingly, the phosphorylation-defective NSP1 E486Q mutant did not form a complex with Cul3, supporting a hypothesis that phosphorylation of the NSP1 ILD triggers structural changes that must occur for the protein to bind Cul3. Whether the putative structural change results from just the phosphorylation of the ILD or also requires the binding of β-TrCP remains to be addressed. The NSP1 RING mutant C42A failed to form a complex with the Cul3 complex, pointing to a key role for the RING in the incorporation of NSP1 into a CRL. The RING domain, including its C42 residue, is conserved among the NSP1 proteins of group A rotaviruses, regardless of whether the protein targets β-TrCP or some other protein (e.g., IRF3) (32). The same RING domain is present in group C, D, and F rotaviruses (33), suggesting that the RING is a highly conserved structural feature necessary for formation of an NSP1-Cul3 CRL. Although the conserved RING domain suggests that the group C, D, and F rotaviruses incorporate into Cul3 CRLs like the group A rotaviruses, the group C, D, and F viruses lack a C-terminal ILD and thus are likely to target host proteins other than β-TrCP. The rotavirus RING domain is known to coordinate zinc binding (34); it may be the loss of this function and the subsequent failure to form zinc-stabilized fingers that prevent the NSP1 C42A mutant from binding to Cul3. There have been multiple reports indicating that NSP1 is a single-stranded RNA-binding protein, with this activity mapping to the RING domain (34, 35). Thus, NSP1 is likely a multifunctional protein with RING residues that contribute to more than one process.

Many animal group A rotaviruses (e.g., SA11-4F, RRV, EW, K9) encode NSP1 proteins that target IRF3 and other IRF proteins for degradation, instead of β-TrCP (4). The NSP1 of these viruses have a conserved C-terminal LL(I/L)S motif that mediates recognition of IRF3. The NSP1 LL(I/L)S motif mimics the recognition motif ρLxIS (ρ is hydrophobic) used by adaptor proteins of the IFN signaling pathway (STING, MAVS, and TRIF) to recognize IRF3 (36). Interestingly, the interaction of the adaptor protein with IRF3 requires phosphorylation of the serine residue in the ρLxIS recognition motif by IKK kinase. In contrast, phosphorylation of the LL(I/L)S motif is not required for interaction of animal NSP1 proteins with IRF3, due to alternative contacts made between NSP1-IRF3 that do not involve the serine residue (37). As a result, NSP1 proteins targeting IRF3 need not undergo the same phosphorylation-dependent activation required by NSP1 proteins targeting β-TrCP. To avoid the assembly of functionless NSP1 CRLs, it is likely that interactions of IRF3 with the C-terminal end of NSP1 would likely be necessary to induce structural changes enabling NSP1 to bind Cul3.

An important observation made in our experiments was that at relatively early times of infection with OSU rotavirus (6 to 8 h p.i.), phosphorylated IκB accumulated despite approximately wild-type levels of β-TrCP. Because β-TrCP is responsible for the degradation of IκB, these results suggested that β-TrCP in the infected cell was rendered inactive without undergoing degradation. This phenomenon could be explained by presuming that NSP1 can interfere with IκB degradation simply by binding, and sequestering, β-TrCP. Alternatively, it could be that NSP1-mediated ubiquitination of β-TrCP is the critical element that prevents β-TrCP and SCF^β-TrCP^ from targeting IκB for ubiquitination and degradation. In this case, NSP1 could render β-TrCP inactive without requiring a long-term sequestering interaction with NSP1.

## MATERIALS AND METHODS

### Cells and viruses.

Human embryonic kidney HEK293T cells were grown in Dulbecco’s modified Eagle’s medium (DMEM) containing 10% fetal bovine serum (FBS; Gibco), 2 mM glutamine, and 1,000 units per ml each of penicillin and streptomycin. Human colorectal HT29 cells were grown in McCoy’s 5A (modified) medium containing 10% FBS and penicillin-streptomycin. African green monkey fetal kidney (MA104) cells were grown in medium M199 containing 10% FBS and penicillin-streptomycin. Porcine PK15 cells were grown in Eagle’s minimal essential medium containing 10% FBS and penicillin-streptomycin. Rotaviruses were propagated, and titers were determined in a plaque assay on MA104 cells, as described by Arnold et al. (38). Prior to infection, rotaviruses were activated by treatment with 5 μg per ml of porcine pancreas trypsin (type IX-S; Sigma-Aldrich) for 1 h at 37°C. The monoreassortant rotavirus strains SOF, SKF, SDF, and SRF were a kind gift of Nobumichi Kobayashi (39).

### Antibodies.

Rabbit polyclonal antibodies to simian SA11-5S and porcine OSU NSP1 proteins (4) and PCNA (sc-7907; Santa Cruz Biotech [SCB]), rabbit monoclonal antibody to β-TrCP (11984S; Cell Signaling Technology, Inc. [CST]), and mouse monoclonal antibodies to IκB (4814; CST), p-IκB (9246; CST), and CKIIα (sc-373894; SCB) were used at a 1:5,000 dilution. Mouse monoclonal antibody to HALO tag (G928A; Promega) and rabbit polyclonal antibodies to Cul1 (A303-373A; Bethyl Lab [BL]), cyclophilin B (D1V5J; CST), and Cul3 (A301-109A; BL) were used at a 1:1000 dilution. Rabbit polyclonal anti-enterokinase cleavage (DDDDK) antibody (A190-102A; BL) was used at a 1:10,000 dilution to detect FLAG tags. Horseradish peroxidase (HRP)-conjugated goat anti-rabbit IgG antibody (7074; CST) and horse anti-mouse IgG antibody (7076; CST) were used at a 1:10,000 dilution.

### Inhibitors.

The protein kinase CKII inhibitor TBB (Tocris Bioscience) and the small-molecule inhibitor of Nedd8-activating enzyme, MLN4924 (Cayman Chemical), were prepared as 1 mM stocks in dimethyl sulfoxide (DMSO) and were stored at −20°C. TBB and MLN4924 were diluted in cell culture medium to final concentrations of 80 and 1 μM, respectively, immediately prior to use. TNF-α (Sigma) was dissolved in water at a concentration of 25 μg/ml and diluted in medium to a final concentration of 25 ng/ml. MG132 (Sigma) was dissolved in DMSO and diluted in medium to a final concentration of 15 μM.

### Plasmids.

The construction of CMV expression vectors pLIC6 and pLIC6F, used for transient expression of wild-type and mutant forms of NSP1 or FLAG-tagged β-TrCP, were described previously (5). pcDNA3-DN-hCUL3-FLAG was a gift from Wade Harper (Addgene plasmid 15820) (3). pHTN-NSP1(OSU) was kindly provided by Michelle Arnold (9). Plasmids were grown in TOP10 *Escherichia coli* cells (Thermo, Fisher), purified using NucleoBond Xtra endotoxin-free plasmid purification kits (Clontech), and verified by sequencing.

### Transfections.

For transient protein expression, transfection mixtures containing 94 µl Opti-MEM (Gibco), 1 μg vector DNA, and 2 µl Lipofectamine 2000 (Invitrogen) were placed in wells of a 12-well cell culture plate. Afterwards, 0.9 ml HEK293T cell suspension (1.3 × 10^6^ cells per ml) in DMEM containing FBS was added to each well. At 24 h posttransfection (p.t.), cell monolayers were rinsed with phosphate-buffered saline (PBS; pH 7.4) and lysed in 200 µl solution containing 1× radioimmunoprecipitation assay (RIPA) buffer (150 mM NaCl, 50 mM Tris-HCl [pH 8.0], 1% Nonidet P-40, 0.5% sodium deoxycholate, 0.1% sodium dodecyl sulfate) and 1× phosphatase inhibitor cocktail (1 mM each of sodium fluoride, sodium orthovanadate, β-glycerophosphate, and sodium pyrophosphate) and 1× Complete EDTA-free protease inhibitor cocktail (Roche). Lysates were incubated on ice for 30 min with vortexing every 10 min and clarified by centrifugation for 10 min at 15,000 × *g* at 4°C.

For RNA interference experiments, CKII-directed siRNA (CSNK2A1 siRNA, L-003475-00; Dharmacon SMARTpool ON-TARGETplus) or scrambled control siRNA (D-001810-10; ON-TARGETplus nontargeting pool) was added to siLentFect (1703360; BioRad) transfection mixtures to a final concentration of 10 or 50 nM, along with 1 μg of plasmid DNA, according to the manufacturer’s instructions. Lysates were prepared at 48 h p.t. and analyzed by immunoblot assay.

### Viral infection.

Nearly confluent monolayers of HT29 cells in 10-cm^2^ tissue culture dishes were infected at a multiplicity of infection (MOI) of 5 with trypsin-activated rotavirus. After a 1-h adsorption period, virus inoculum was removed and cell monolayers were rinsed with PBS. Cell monolayers were then maintained in serum-free McCoy’s 5A (modified) medium until time of harvest. Cell lysates were prepared by washing monolayers with cold PBS and scraping cells into 0.5 ml RIPA lysis buffer. Lysates were incubated on ice for 10 min, gently mixed, and clarified by centrifugation for 10 min at 15,000 × *g* at 4°C.

### Immunoblot assay.

Protein samples were mixed with NuPAGE-LDS sample buffer (Invitrogen) containing 50 mM dithiothreitol (DTT), then denatured by heating to 70°C for 10 min and resolved by electrophoresis on precast 10% Tris–glycine polyacrylamide gels (Novex). Molecular weight markers (SeeBlue Plus2 [Invitrogen] and EZ-Run [Fisher Scientific]) were resolved in parallel to determine protein sizes. Proteins were transferred from gels onto nitrocellulose membranes by using an iBlot dry transfer apparatus (Thermo, Fisher). Membranes were blocked by incubation in 5% Carnation dry milk dissolved in PBS–0.2% Tween 20 (PBST) prior to incubation with primary antibody diluted in milk-PBST solution. Membranes were washed with PBST before incubating with HRP-conjugated secondary antibody diluted in milk-PBST solution. Membranes were washed with PBST and developed using the SuperSignal West Pico chemiluminescent substrate (Pierce). Signal was detected by exposing membranes to BioExcell X-ray film or by using the Azure Series c500 infrared imaging system. Bands of interest were normalized to the loading control, PCNA.

### In-blot phosphatase treatment.

Protein samples were resolved by electrophoresis on 10% polyacrylamide gels and transferred onto nitrocellulose membranes, as described above. Membranes were blocked by incubation at room temperature in 5% bovine serum albumin dissolved in PBST. Afterwards, membranes were incubated overnight at room temperature in a solution containing 100 units per ml of CIP (NE M0290S), 100 mM NaCl, 50 mM Tris-HCl (pH 7.9), 10 mM MgCl_2_, and 1 mM DTT. Following an additional 30-min incubation at 37°C, membranes were rinsed with PBST and probed by immunoblot assay.

### Immunoprecipitation.

Nearly confluent monolayers of HEK293T cells in 10-cm^2^ dishes containing 10 ml of medium were transfected by adding plasmid DNA mixtures prepared from 1.4 ml Opti-MEM, 7.5 µg each of pLIC6-NSP1 and pLIC6F-β-TrCP or pcDNA3-DN-hCUL3-FLAG, and 29 µl Lipofectamine 2000. Transfected cells were incubated for 24 h at 37°C, harvested by resuspending into medium, and recovered by low-speed centrifugation (5 min at 500 × *g*). Cell pellets were rinsed twice with cold PBS and disrupted by resuspension in immunoprecipitation (IP) lysis buffer (150 mM NaCl, 50 mM Tris-HCl [pH 7.4], 1% Triton X-100, with 1× Complete EDTA-free protease inhibitor cocktail). After incubation on ice for 1 h, cellular debris were removed by centrifugation at 14,000 × *g* for 10 min. Fifty microliters of a slurry of anti-FLAG M2 magnetic beads (M8823; Sigma), prerinsed 3 times with IP wash buffer (150 mM NaCl, 50 mM Tris-HCl [pH 7.4]), was added to clarified cell lysates. After overnight incubation at 4°C with nutation, M2 beads were recovered using a BioRad Surebeads magnetic stand and then rinsed 3 times with IP wash buffer. Protein was released from M2 beads by nutation in IP elution buffer (0.1 M glycine [pH 3.0]) for 5 min at 4°C. Eluted proteins were examined by gel electrophoresis and immunoblot analysis.
